# Pro-Inflammatory Dietary Profiles Are Associated with Psychosocial Functioning in Spanish Adolescents: Findings from the EHDLA Study

**DOI:** 10.3390/nu18183073

**Published:** 2026-09-20

**Authors:** Paula Marrero-Fernández, Jacinto Muñoz-Pardeza, Camila Miño, José Francisco López-Gil, Miguel López-Moreno

**Affiliations:** 1 Department of Chemical Engineering and Pharmaceutical Technology, University of La Laguna, Avda. Astrofísico Francisco Sánchez s/n, 38001 Santa Cruz de Tenerife, Spain; 2 Navarrabiomed, Hospital Universitario de Navarra (HUN), Universidad Pública de Navarra (UPNA), Instituto de Investigación Sanitaria de Navarra (IdiSNA), 31008 Pamplona, Spain; 3 European Institute of Higher Studies (IEES), 4820-509 Fafe, Portugal; 4 School of Medicine, Universidad Espíritu Santo, Samborondón 092301, Ecuador; 5 Faculty of Health Sciences, Universidad Autónoma de Chile, Temuco 4810101, Chile; 6 Diet, Planetary Health and Performance, Faculty of Health Sciences, Universidad Francisco de Vitoria, 28223 Madrid, Spain; miguel.lopez@ufv.es; 7 Institute of Health and Sport Sciences, Faculty of Health Science, Universidad Francisco de Vitoria, 28223 Madrid, Spain

**Keywords:** dietary inflammatory index, diet-related inflammation, behavioral problems, adolescents, psychosocial functioning, strengths and difficulties questionnaire

## Abstract

**Background:** Dietary factors may influence psychosocial outcomes through inflammatory pathways, but evidence in adolescents remains limited. The aim was to examine whether dietary inflammatory potential is associated with psychosocial functioning among Spanish adolescents. **Methods:** This cross-sectional study is based on a subsample from the Eating Healthy and Daily Life Activities (EHDLA) project, conducted in the Region of Murcia (Spain). Dietary inflammatory potential was assessed using a dietary inflammatory index (DII)-informed Food Frequency Questionnaire (FFQ)-derived inflammatory score based on a validated 45-item food frequency questionnaire. Behavioral functioning was evaluated using the Strengths and Difficulties Questionnaire (SDQ). Associations between DII-informed FFQ-derived inflammatory scores and SDQ outcomes were examined using robust linear regression models adjusted for sociodemographic, lifestyle, and anthropometric factors. **Results:** The analysis included 674 adolescents (57.1% girls; age: 14.0 [13.0–15.0] years). Higher DII-informed FFQ-derived inflammatory score (more pro-inflammatory diet) was associated with greater total behavioral difficulties (unstandardized beta coefficient [B] = 0.058; 95% confidence interval [CI]: 0.005–0.111; standardized β = 0.099), conduct problems (B = 0.025; 95% CI: 0.011–0.039; β = 0.148), hyperactivity/inattention (B = 0.032; 95% CI: 0.013–0.052; β = 0.154), and externalizing problems (B = 0.056; 95% CI: 0.028–0.084; β = 0.181), and with lower prosocial behavior (B = −0.029; 95% CI: −0.045 to −0.014; β = −0.149). Multiple imputation sensitivity analyses yielded estimates in a similar direction, although the associations did not reach statistical significance. No significant associations were observed for emotional symptoms, peer relationship problems, or internalizing difficulties. **Conclusions:** Higher dietary inflammatory potential was associated with a less favorable psychosocial functioning profile among adolescents, particularly in externalizing domains. Longitudinal studies and anti-inflammatory dietary interventions are needed to determine whether the observed associations translate into meaningful changes in psychosocial functioning.

## 1. Introduction

Dietary factors have increasingly been recognized as potential contributors to psychological outcomes, extending beyond their traditional role in physical health [1,2,3]. Among the proposed mechanisms, low-grade systemic inflammation has emerged as a biologically plausible pathway linking nutrition to behavioral outcomes in adulthood [4,5]. In adult populations, more pro-inflammatory dietary patterns have been associated with poorer psychosocial or behavioral outcomes, particularly depressive symptoms and psychological distress [6]. However, the extent to which the inflammatory potential of the diet relates to psychosocial functioning during earlier developmental stages remains insufficiently understood.

Adolescence constitutes a particularly sensitive developmental period characterized by high neuroplasticity and the progressive maturation of executive and social functions. Epidemiological evidence indicates that approximately 50% of adult mental health disorders develop during adolescence [7]. During this stage, environmental exposures, including diet, may exert lasting effects on behavioral development, although the specific role of dietary inflammatory potential remains largely unexplored in this population [8]. However, the specific contribution of dietary exposures during adolescence, as distinct from those occurring earlier in life, remains unclear. This highlights the importance of identifying modifiable early-life determinants of psychosocial functioning. Previous studies have reported associations of poorer diet quality with increased emotional and behavioral problems in children [9,10]. However, most of these studies rely on general diet quality indices, which may not capture the cumulative inflammatory potential of dietary patterns and their underlying biological mechanisms linking diet to behavior.

Specific dietary components can promote or suppress inflammation: saturated fats and refined carbohydrates tend to promote pro-inflammatory responses, whereas dietary fiber, polyphenols, and certain micronutrients may exert anti-inflammatory effects [11]. Consequently, capturing the overall inflammatory potential of dietary exposures has become increasingly relevant for understanding diet–behavior relationships. The Dietary Inflammatory Index (DII) literature provides a validated, literature-derived scoring framework that quantifies the inflammatory properties of an individual’s diet based on multiple food components, each weighted by their documented effects on inflammatory biomarkers [12]. In adult populations, higher DII-informed Food Frequency Questionnaire (FFQ)-derived inflammatory scores (indicating more pro-inflammatory diets) have been consistently associated with poorer psychosocial functioning, including depressive symptoms and psychological distress [12,13,14]. In contrast, evidence in pediatric populations remains limited, particularly regarding psychosocial and social functioning assessed with comprehensive instruments.

To address this gap, validated instruments such as the Strengths and Difficulties Questionnaire (SDQ) allow for a multidimensional assessment of psychosocial functioning, capturing both strengths (e.g., prosocial behavior) and difficulties (e.g., emotional symptoms, conduct problems, hyperactivity, and peer relationship problems) [15]. This study aimed to evaluate whether the inflammatory potential of the diet, quantified using the DII-informed FFQ-derived inflammatory score, is associated with psychosocial functioning in Spanish adolescents, assessed through the SDQ. We hypothesized that higher DII-informed FFQ-derived inflammatory score (more pro-inflammatory diets) would be associated with greater behavioral difficulties, including higher total difficulties, conduct problems, hyperactivity/inattention, and externalizing problems, as well as lower prosocial behavior, adjusted for potential confounders (sociodemographic, lifestyle, and anthropometric factors).

## 2. Materials and Methods

### 2.1. Study Design and Population

This cross-sectional study was reported in accordance with the Strengthening the Reporting of Observational Studies in Epidemiology (STROBE) guidelines (Supplementary STROBE checklist in Table S1). We used data from adolescents participating in the Eating Healthy and Daily Life Activities (EHDLA) study, conducted during the 2021–2022 academic year [16]. The study was carried out across all three secondary schools located in the Valle de Ricote (Region of Murcia, Spain), with data collection performed during scheduled physical education classes. These three schools constitute the public secondary-school population of this region. Selection was based on geographic coverage of the study catchment area rather than random sampling of schools. Participants were eligible if they (1) were aged between 12 and 17 years, (2) provided written informed consent from both themselves and their legal guardians, and (3) were enrolled in or resided within the Valle de Ricote. Exclusion criteria included: (1) exemption from Physical Education classes at school, as both questionnaire administration and study assessments were conducted during these sessions; (2) the presence of any medical condition that contraindicated physical activity or required special attention; (3) current pharmacological treatment; (4) lack of parental or legal guardian consent; and (5) refusal of the adolescent to participate in the study. Further methodological details of the EHDLA study have been previously described elsewhere [16]. Participants did not receive any financial compensation, gifts, or other incentives for participating in the study.

The study adhered to the Declaration of Helsinki and received approval from the Ethics Committee of the Albacete University Hospital Complex (ID 2021-85) and the Bioethics Committee of the University of Murcia (ID 2218/2018). Examples of medical conditions that contraindicated physical activity or required special attention included acute musculoskeletal injuries precluding participation in Physical Education, unstable cardiovascular conditions, and severe musculoskeletal or neurological conditions requiring adapted Physical Education or individualized medical supervision during physical activity. These criteria were ascertained through parental/legal-guardian informed consent procedures, school Physical Education eligibility (including exemption status), and information provided to research staff before assessments.

Figure S1 summarizes participant flow from the EHDLA study (*N* = 1378) to analytical samples (*n* = 674 unadjusted; *n* = 629 adjusted). The percentage of missing data for each variable is presented in Supplementary Table S2. For the main analyses, complete cases were used (*n* = 629 for adjusted models). The proportion of missing data ranged from 8.5% (Body Mass Index, BMI) to 46.4% (SDQ) in the full cohort.

### 2.2. Study Variables

All questionnaires were administered during scheduled Physical Education classes under the supervision of trained research staff. Prior to data collection, researchers received standardized training regarding study procedures, questionnaire administration, anthropometric assessments, and participant instructions. To ensure data quality, standardized protocols were followed across all schools, participants were encouraged to ask questions when clarification was needed, and completed questionnaires were reviewed on-site for completeness before data entry. Questionnaires were administered in paper format.

#### 2.2.1. Psychosocial Functioning

Psychosocial functioning was assessed using the self-reported SDQ [15], a validated instrument for evaluating emotional, behavioral, and social functioning among adolescents aged 11–17 years. The SDQ includes five subscales: emotional symptoms, conduct problems, hyperactivity/inattention, peer relationship problems, and prosocial behavior, each ranging from 0 to 10 points. In addition to the individual subscales, composite scores were calculated to assess broader psychosocial domains. Responses were rated on a three-point scale (0 = not true, 1 = somewhat true, 2 = certainly true). The Spanish self-report version of the SDQ has demonstrated acceptable internal consistency in Spanish adolescents, with a McDonald’s omega of 0.74 for the Total Difficulties score and values ranging from 0.52 to 0.71 across the five subscales. Evidence of validity based on the internal structure has also supported the five-factor model of the SDQ, with factor loadings ranging from 0.35 to 0.78 [17].

A total difficulties score was calculated by summing all subscales except prosocial behavior. Externalizing problems were derived from conduct problems and hyperactivity scores, whereas internalizing problems were calculated by summing emotional symptoms and peer problems. Both derived scores ranged from 0 to 20 points.

#### 2.2.2. DII-Informed FFQ-Derived Inflammatory Score

Dietary intake was assessed through a self-administered Food Frequency Questionnaire (FFQ) validated for Spanish populations [18]. The FFQ assessed habitual dietary intake by recording the frequency of consumption of 45 food items during a typical period, with responses reported as times per week or month. Following the validated protocol, consumption frequencies were converted into estimated daily intakes using standardized portion sizes. The French food composition table Répertoire général des aliments (REGAL) was used to calculate total energy intake [19]. Nutrient intakes were subsequently estimated using the Spanish Food Composition Database (BEDCA) and standardized portion sizes from the validated FFQ protocol. Participants reported the habitual frequency of consumption of a wide range of foods, which were subsequently used to estimate both total energy intake and the inflammatory potential of the diet. The inflammatory potential of dietary intake was evaluated using a DII-informed FFQ-derived inflammatory score, a literature-derived scoring system developed to quantify the overall inflammatory properties of dietary exposures based on their associations with circulating inflammatory biomarkers [20]. The DII-informed FFQ-derived inflammatory score incorporates multiple dietary parameters, including macronutrients, micronutrients, and specific food components, each weighted according to their documented pro- or anti-inflammatory effects. Higher DII-informed FFQ-derived inflammatory scores indicate more pro-inflammatory dietary patterns, whereas lower (more negative) scores reflect more anti-inflammatory dietary profiles. Detailed information regarding the development and validation of the DII has been published previously [20]. For the present study, dietary inflammatory potential was operationalized using a DII-informed FFQ-derived inflammatory score based on the 45 FFQ food items. Each item was assigned a directional inflammatory weight informed by published DII evidence on corresponding food groups/nutrients (Supplementary Table S3). The score equals the sum of daily servings multiplied by item-specific weights. This food-item proxy should not be interpreted as the canonical nutrient-based globally standardized DII. Following the conceptual framework of the DII, each of the 45 FFQ food items was assigned an a priori inflammatory direction/weight based on its nutrient profile and reported associations with inflammatory biomarkers (e.g., C-reactive protein (CRP), interleukin 6 (IL-6), tumor necrosis factor α (TNF-α)). Inflammatory weights ranged from −0.8 (most anti-inflammatory) to 0.6 (most pro-inflammatory) [21]. The score was computed as Σ (daily servings_i_ × weight_i_). The same DII-informed FFQ-derived scoring approach has been previously applied in the EHDLA study [21]. The present food-based operationalization was selected to characterize the inflammatory potential of the dietary information captured by the 45-item FFQ. Exact numerical weights were adopted from the previous EHDLA application of this food-based score [21] and are tabulated in Supplementary Table S3. This weighting system has not been formally validated against inflammatory biomarkers or the canonical nutrient-based DII. Accordingly, the score should be interpreted as a pragmatic proxy.

### 2.3. Covariates

Information on age and sex was collected through self-report. Socioeconomic status was assessed using the Family Affluence Scale-III (FAS-III) [22]. Weight (kg) and height (cm) were measured following standardized procedures, and BMI was calculated as kg/m^2^. Total energy intake was derived from the FFQ. Physical activity and sedentary behavior were evaluated using the Spanish version of the Youth Activity Profile (YAP-S) [23]. Both variables were expressed as questionnaire-derived YAP-S scores (unitless), with higher scores indicating greater physical activity and sedentary behavior, respectively. Habitual sleep duration (hours) was estimated using self-reported bedtimes and wake-up times provided separately for weekdays and weekends [24,25]. An average weekly sleep duration was calculated by weighting weekday sleep duration by five days and weekend sleep duration by two days, summing both values and dividing the total by seven to obtain a standardized estimate [16]. These covariates were selected a priori based on prior literature indicating their potential relevance for adolescent psychosocial health outcomes. Formal directed acyclic graph-based selection was not performed because family history of mental health problems, maternal diet during pregnancy, and early childhood nutrition were not available in EHDLA [26,27,28,29].

### 2.4. Statistical Analysis

All analyses were conducted using R statistical software (version 4.4.0), along with RStudio (2024.04.1 + 748). Normality of continuous variables was assessed using quantile–quantile plots, density plots, and the Shapiro–Wilk test. Continuous variables were expressed as medians and interquartile ranges (IQRs), whereas categorical variables were presented as frequencies and percentages (%).

Associations between the DII-informed FFQ-derived inflammatory score and SDQ outcomes were evaluated using linear regression models to account for heteroscedasticity and potential outliers. Specifically, robust regression was performed using the ‘lmrob’ function from the ‘robustbase’ package (‘KS2014’ setting) [30]. Both unadjusted and adjusted models were estimated. Adjusted models included age, sex, socioeconomic status (FAS-III score), BMI, sleep duration, physical activity (YAP-S score), sedentary behavior (YAP-S score), and total energy intake as covariates. Unstandardized beta coefficient (B) and 95% confidence interval (CI) were reported. Standardized beta coefficients (β) were reported to facilitate comparison of effect sizes across outcomes. The magnitude of standardized coefficients was interpreted using conventional thresholds (|β| = 0.10–0.29 small, 0.30–0.49 moderate, and ≥0.50 large) [31,32].

The target estimate was the conditional mean difference in each continuous SDQ score per one-unit increase in the DII-informed FFQ-derived inflammatory score. Sensitivity analyses included minimally adjusted models (age, sex, FAS-III), scaled associations per 1-SD and IQR increase (Supplementary Table S4), quasi-Poisson regression (Supplementary Table S5), and minimal versus full adjustment comparisons (Supplementary Table S6). SDQ Total Difficulties were the prespecified primary outcome; subscale/composite analyses were explored with false discovery rate (FDR).

To evaluate the potential impact of missing data, we report missingness by variable, participant flow, and a comparison of included and excluded participants. Additional sensitivity analyses compared robust linear (primary) and ordinary least squares regression models, summarized robust regression downweighting, applied Benjamini–Hochberg false-discovery-rate correction across the eight SDQ outcomes, and calculated E-values for significant adjusted associations to assess robustness to unmeasured confounding [33]. Statistical significance was set at α = 0.05 (reject when *p* < 0.05). Because a substantial fraction of participants had incomplete SDQ and/or covariate data, we did not assume missing completely at random (MCAR) as a default. As a working assumption for sensitivity analyses, missingness was treated as missing at random (MAR) conditional on observed dietary, sociodemographic, lifestyle, and anthropometric variables [34]. Little’s MCAR test: statistic = 153.54, *p* = 0.003. MCAR rejected; MAR used as working assumption for MI; MNAR/selection bias cannot be excluded. Primary adjusted analyses remained complete-case (*n* = 629). As an additional sensitivity analysis, multiple imputation by chained equations (MICE) was performed among diet-eligible participants after excluding inflammatory-score outliers (*N* = 928). The exposure, SDQ outcomes, and covariates were imputed, and estimates were subsequently pooled using Rubin’s rules (Supplementary Table S7). Alongside the exposure, SDQ outcomes, and covariates, auxiliary variables included family structure, height, weight, and individual FAS-III items (when available); continuous variables were imputed using predictive mean matching (m = 20; maxit = 10). Chain-mean traces were inspected for convergence (Supplementary Figure S2). The fraction of missing information for the exposure coefficient ranged from 0.511 to 0.703 across SDQ outcomes (Supplementary Table S8), consistent with the substantial incomplete-case burden and supporting cautious interpretation of the MI estimates. Descriptive data for individual FAS-III items are provided in Supplementary Table S9. Within the analytical sample (*n* = 674), missingness for covariates used in adjusted models was low (e.g., BMI 5.5%; FAS-III 1.0%; sleep 1.2%; YAP-S 1.3%).

## 3. Results

The descriptive characteristics of the study sample are presented in Table 1. The final analytical sample included 674 adolescents. For adjusted models, complete-case analyses were performed on 629 participants with data available for all covariates. The median age was 14.0 years (IQR: 13.0–15.0), and 57.1% were girls. The median BMI was 21.6 kg/m^2^ (IQR: 19.3–25.1). Median daily energy intake was 2582.0 kcal/day (IQR: 1954.5–3460.5), and median sleep duration was 8.3 h/day (IQR: 7.6–8.8). The median YAP-S physical activity score was 2.6 (IQR: 2.2–3.0), and the median YAP-S sedentary behavior score was 3.4 (IQR: 2.8–4.0).

The DII-informed FFQ-derived inflammatory score was 0.3 (IQR: −5.4 to 6.6). Regarding psychosocial functioning, the median total difficulties score from the SDQ was 11.0 (IQR: 7.0–16.0). Median scores for SDQ subscales were 3.0 (IQR: 1.0–5.0) for emotional symptoms, 2.0 (IQR: 1.0–3.0) for conduct problems, 4.0 (IQR: 3.0–6.0) for hyperactivity/inattention, 2.0 (IQR: 1.0–3.0) for peer relationship problems, and 8.0 (IQR: 7.0–9.0) for prosocial behavior.

Associations between DII-informed FFQ-derived inflammatory scores and SDQ outcomes are presented in Table 2.

After adjustment for age, sex, socioeconomic status, BMI, sleep duration, physical activity, sedentary behavior, and total energy intake (*n* = 629, complete-case analysis), a higher DII-informed FFQ-derived inflammatory score was associated with higher total difficulties (B = 0.058; 95% CI: 0.005 to 0.111; *p* = 0.031; β = 0.099). Regarding the composite SDQ domains, higher DII-informed FFQ-derived inflammatory scores were associated with greater conduct problems (B = 0.025; 95% CI: 0.011 to 0.039; *p* < 0.001; β = 0.148), hyperactivity/inattention (B = 0.032; 95% CI: 0.013 to 0.052; *p* = 0.001; β = 0.154), and externalizing problems (B = 0.056; 95% CI: 0.028 to 0.084; *p* < 0.001; β = 0.181). In contrast, a higher DII-informed FFQ-derived inflammatory score was associated with lower prosocial behavior scores (B = −0.029; 95% CI: −0.045 to −0.014; *p* < 0.001; β = −0.149). No significant associations were identified for emotional symptoms, peer relationship problems, or internalizing problems in adjusted analyses. Standardized effect sizes ranged from β = 0.099 to β = 0.181, corresponding to small effect sizes according to conventional thresholds. After Benjamini–Hochberg correction across the eight SDQ outcomes, associations for conduct problems, hyperactivity/inattention, prosocial behavior, and externalizing problems remained statistically significant (adjusted *p* < 0.05), whereas the association for total difficulties was at the significance threshold (adjusted *p* = 0.050) (Supplementary Table S10). Sensitivity analyses using Ordinary Least Squares (OLS) models yielded directionally consistent estimates (Supplementary Table S11), and downweighting of potential outliers in robust regression was limited (Supplementary Table S12). E-values suggested limited robustness to unmeasured confounding for the significant associations (Supplementary Table S13). Participants included in adjusted analyses were broadly comparable to those excluded on age, sex, BMI, socioeconomic status, and energy intake (Supplementary Table S14). In a further sensitivity analysis retaining the eight inflammatory-score outliers (adjusted *N* = 634), associations remained directionally consistent with the primary complete-case models: total difficulties (B = 0.061; 95% CI: 0.019 to 0.103; *p* = 0.004); conduct problems (B = 0.017; 95% CI: 0.006 to 0.028; *p* = 0.003); hyperactivity/inattention (B = 0.031; 95% CI: 0.015 to 0.046; *p* < 0.001); externalizing problems (B = 0.046; 95% CI: 0.024 to 0.069; *p* < 0.001); prosocial behavior (B = −0.013; 95% CI: −0.025 to 0.000; *p* = 0.051) (Supplementary Table S15). In multiple-imputation sensitivity analyses (MICE; diet-eligible sample *N* = 928), pooled estimates remained directionally consistent with the complete-case findings, although none of the associations reached statistical significance: total difficulties (B = 0.04; 95% CI: −0.01 to 0.09; *p* = 0.099), externalizing problems (B = 0.04; 95% CI: −0.001 to 0.08; *p* = 0.054), and prosocial behavior (B = −0.03; 95% CI: −0.05 to 0.0003; *p* = 0.053) (Supplementary Table S7). Per 1-SD increase (SD = 10.2), total difficulties B = 0.60 (95% CI: 0.05 to 1.14); per IQR (11.6 units), B = 0.68 (95% CI: 0.06 to 1.29) (Supplementary Table S4). Minimal models were directionally consistent (Supplementary Table S6).

**Figure 1:**
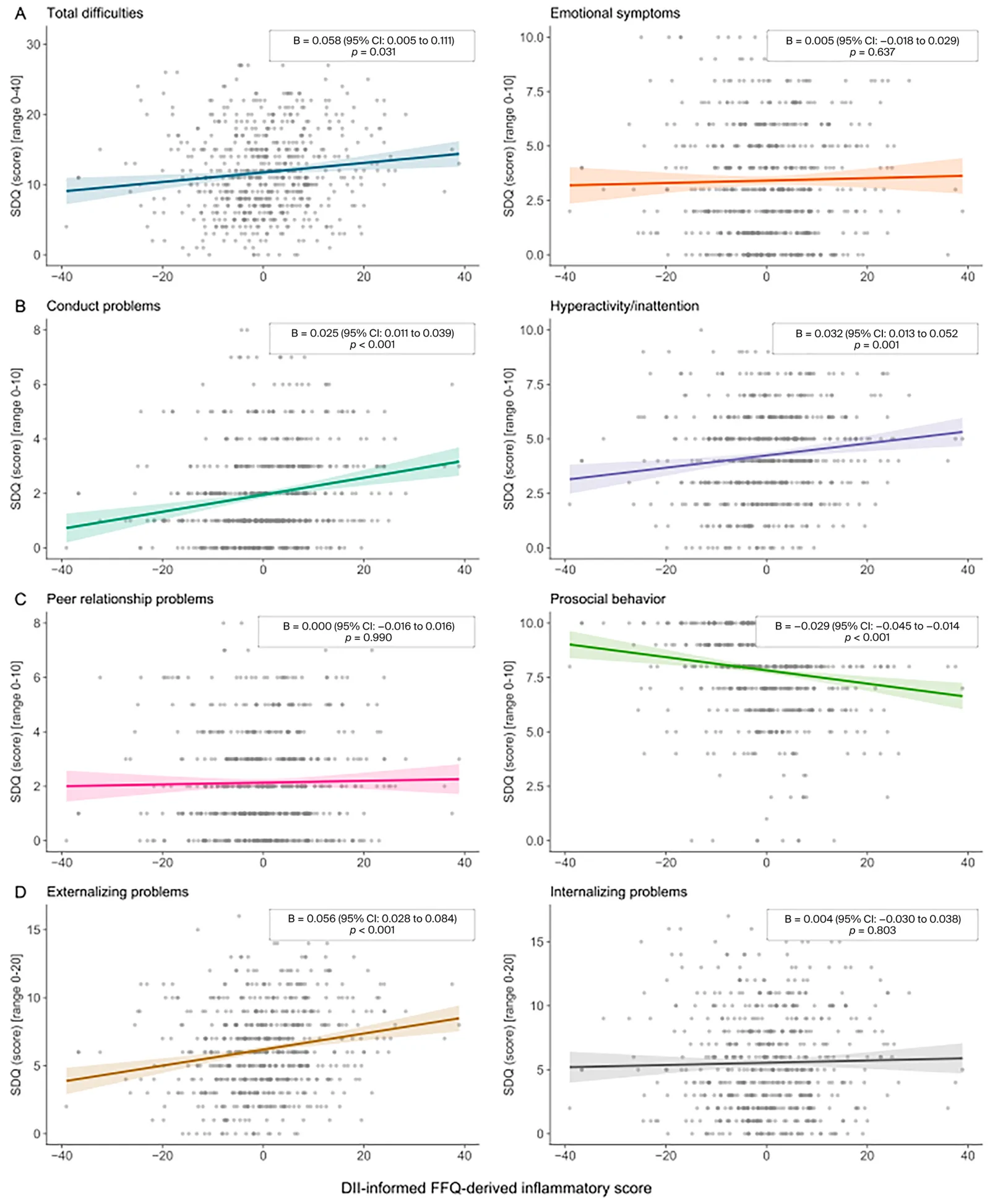
Visual inspection of adjusted associations between the DII-informed FFQ-derived inflammatory score and SDQ outcomes (panels (**A**–**D**); two outcomes per panel). Gray dots represent individual observations; the colored line and shaded band show the linear fit and its 95% confidence interval, respectively, while the B and p-value in each subplot correspond to this unadjusted fit shown for visual reference only (see Table 2 for adjusted estimates).

Figure 1 presents the graphical representation of the adjusted associations between the inflammatory score and SDQ outcomes. Scatter plots with linear fit lines and 95% confidence bands are shown for visual inspection only; inferential estimates were obtained from robust regression models (Table 2). Supplementary Figures S3–S10 display the residual diagnostics for total SDQ and each SDQ outcome.

## 4. Discussion

In this study, we found that a higher dietary inflammatory potential was associated with less favorable psychosocial functioning in Spanish adolescents, primarily reflected in externalizing domains and lower prosocial behavior. These findings partially support our initial hypothesis, as associations were observed for externalizing difficulties such as conduct problems and hyperactivity/inattention, but not for internalizing domains. Overall, the results suggest that higher dietary inflammatory potential may be associated with psychosocial functioning, particularly externalizing domains and prosocial behavior. Although multiple-imputation analyses yielded estimates in the same direction as the complete-case analyses, the associations with total difficulties, externalizing problems, and prosocial behavior did not reach statistical significance. Therefore, the magnitude and robustness of these associations should be interpreted cautiously. Despite the small observed effect sizes, behavioral outcomes are multifactorial in nature, and individual determinants would not be expected to account for a large proportion of the variance. Therefore, these findings should not be interpreted as indicating clinically meaningful differences at the individual level. Nevertheless, even limited associations may be relevant from a public health perspective when considered alongside other potentially modifiable influences on adolescent psychosocial functioning. From a broader perspective, these findings support the relevance of dietary inflammatory potential as a correlate of psychosocial functioning.

To our knowledge, this is the first study to specifically examine the association between dietary inflammatory potential and SDQ outcomes in adolescents. Previous studies, including those within the EHDLA cohort, have focused on overall dietary patterns (e.g., Mediterranean diet), which may partly reflect anti-inflammatory properties but do not directly capture the inflammatory potential of the diet. These have shown that lower adherence to this dietary pattern was associated with higher SDQ total difficulties, conduct problems, and hyperactivity, together with lower prosocial behavior [35], closely mirroring the direction observed in the present study. Similarly, a Norwegian study reported that lower adherence to the Norwegian dietary recommendations was associated with higher SDQ total difficulties and hyperactivity, although no association was observed with conduct problems and prosocial behavior was not assessed [36]. In the Raine Study, a Western dietary pattern during adolescence, characterized by higher intake of takeaway foods, confectionery, and red meat, was associated with poorer psychosocial outcomes, including higher internalizing and externalizing problem scores [37]. Similarly, findings from the Research with East London Adolescents: Community Health Survey (RELACHS) cohort of adolescents in the United Kingdom indicated that poorer diet quality was associated with a higher likelihood of psychosocial difficulties measured using the SDQ. These findings are conceptually consistent with our results, as less healthy dietary profiles often reflect dietary patterns with greater inflammatory potential [38]. More broadly, a recent systematic review and meta-analysis also found that greater adherence to the Mediterranean diet was consistently associated with higher health-related quality of life among children and adolescents, further supporting the relevance of overall diet quality for psychosocial well-being in young populations [39].

Longitudinal studies suggest that these associations may emerge early in life and persist over time. In the *Étude sur les déterminants pré- et postnatals du développement et de la santé de l’enfant* (EDEN) cohort, dietary patterns at age 2 predicted trajectories of hyperactivity/inattention and conduct problems between ages 3 and 8 years, assessed using the SDQ, suggesting that dietary exposures early in life may be associated with subsequent behavioral development [40]. Extending this developmental perspective to the prenatal period, a large individual participant data meta-analysis of four European cohorts reported that a more pro-inflammatory maternal diet during pregnancy was associated with a higher risk of offspring emotional and behavioral symptoms, including aggressive behavior and attention deficit hyperactivity disorder (ADHD)-related symptoms [41]. These findings suggest a consistent pattern across different populations and developmental stages, supporting the hypothesis that dietary inflammatory potential may be linked to psychosocial functioning from early life onwards. This life-course perspective is particularly relevant, as early alterations in psychosocial regulation and social functioning may track into later stages of life, potentially influencing academic performance, social relationships, and health-related behaviors. However, because dietary inflammatory potential was assessed only during adolescence, it remains unclear whether the observed associations reflect current or earlier-life dietary exposures.

Population-based biomarker analyses further support the biological plausibility of this framework. Data from the National Health and Nutrition Examination Survey (NHANES) in children and adolescents have linked higher Children’s Dietary Inflammatory Index (C-DII) scores to inflammatory biomarkers [42], indicating that the DII-informed FFQ-derived inflammatory scores may capture meaningful variation in systemic inflammatory status in youth. One proposed mechanism is that diets with higher inflammatory potential may be associated with chronic low-grade inflammation, reflected in elevations of inflammatory biomarkers such as C-reactive protein (CRP) and pro-inflammatory cytokines (e.g., interleukin-6, tumor necrosis factor-α) [43]. Consistent with this hypothesis, a systematic review in children and adolescents reported that healthier dietary patterns tend to be associated with lower levels of inflammatory markers, such as CRP [44].

Inflammatory signaling has been hypothesized to influence behavior through several neurobiological pathways. Pro-inflammatory cytokines can affect neurotransmitter systems, including dopamine and serotonin metabolism and can impair neuroplasticity, particularly in brain regions involved in emotion regulation and behavioral control, such as the prefrontal cortex and limbic structures [45]. Diet has also been proposed to influence behavior through the gut–brain axis, as dietary patterns can shape gut microbiota composition and intestinal barrier function, thereby contributing to immune activation and low-grade inflammation that may influence brain function and behavior [45,46]. These mechanisms may offer one possible explanation for the observed associations between pro-inflammatory dietary profiles and behavioral difficulties in adolescents, suggesting that inflammatory processes could potentially represent one pathway underlying the observed associations between diet and psychosocial functioning. It is also important to acknowledge that this relationship may be bidirectional: Poor psychosocial functioning, particularly hyperactivity and conduct problems, may itself drive poor dietary choices through impaired impulse control, preference for highly palatable energy-dense processed foods, and disrupted family eating routines. This bidirectional dynamic renders reverse causality a plausible alternative explanation that cannot be excluded in cross-sectional designs [47]. Because inflammatory biomarkers were not measured in the present study, the involvement of inflammatory pathways remains speculative.

The selective pattern of associations (involving externalizing but not internalizing outcomes) merits specific attention. In adult populations, the most consistent DII-mental health associations concern depressive symptoms, which are predominantly internalizing [48,49]. The absence of significant associations with emotional symptoms and peer relationship problems in the present adolescent sample may reflect several possibilities. First, self-reported internalizing symptoms in younger adolescents may have lower reliability relative to externalizing behaviors, which are more behaviorally observable [50]. Second, the neurobiological pathways linking dietary inflammation to impulse control and reward processing (primarily dopaminergic and prefrontal circuits) may be more proximally disrupted than those implicated in emotional symptomatology [51,52]. Third, the SDQ subscales share overlapping variance, and the specificity of associations should be interpreted cautiously. Future studies incorporating biomarker validation and longitudinal designs should examine this differential pattern.

This study has several notable strengths. These include the relatively large sample of adolescents, the use of validated instruments to assess psychosocial functioning outcomes, and the comprehensive adjustment for a range of relevant sociodemographic and lifestyle confounders. In addition, the use of robust regression minimized the influence of outliers and heteroscedasticity. Nevertheless, several limitations should be considered when interpreting the findings. First, the cross-sectional design precludes causal inference. Therefore, the observed associations should not be interpreted as evidence of a causal relationship, and reverse causation cannot be excluded: impulsivity, hyperactivity, or behavioral difficulties may themselves influence dietary choices. Longitudinal studies are required to clarify the temporal direction of the observed associations and determine whether dietary modifications can improve psychosocial functioning. Second, a substantial proportion of the initial sample was excluded due to missing data, particularly for psychosocial functioning outcomes (46.4% missing SDQ in the full cohort), which may have introduced selection bias; although MI sensitivity analyses were directionally consistent with complete-case results, residual selection bias under MNAR cannot be ruled out. Third, the inflammatory exposure was assessed using a DII-informed FFQ-derived inflammatory score rather than a validated nutrient-based index such as the DII. Although nutrient intakes were estimated using the BEDCA database, the 45-item FFQ was primarily designed to capture food-group consumption and did not comprehensively assess all dietary parameters required to reproduce these standardized indices. Therefore, the present score should be interpreted as a pragmatic food-based proxy of dietary inflammatory potential rather than as a direct equivalent of the validated DII-based indices. In addition, we did not perform a formal validation or comparative analysis against a recognized DII-based index, which may limit comparability with studies using validated DII-based measures and biomarker-calibrated approaches. Fourth, although the analyses were adjusted for several relevant sociodemographic, lifestyle, and anthropometric factors, residual confounding cannot be excluded. Several potentially important confounders were not assessed, including psychiatric diagnoses, psychotropic medication use, personality traits, family relationship quality, parental mental health, and early-life dietary exposures. Furthermore, although current pharmacological treatment was an a priori exclusion criterion of the EHDLA protocol, no participant was excluded for this reason in the present analytical sample (*n* = 0). Psychotropic medication use was not reassessed analytically; therefore residual confounding by treated behavioral disorders cannot be excluded. Fifth, several variables were assessed using self-reported measures, which may be subject to recall bias or social desirability bias. Sixth, the study was conducted in adolescents from a specific region in Spain (Valle de Ricote), which may limit generalizability. Seventh, although the FFQ has been validated for estimating energy and nutrient intakes, measurement error inherent to self-reported dietary assessment may have influenced estimation of the inflammatory score. Eighth, although E-values were calculated to explore unmeasured confounding, the observed E-values were close to the null (1.01–1.02 for point estimates), indicating limited robustness to unmeasured confounding. Finally, data collection took place during the academic year immediately following the Coronavirus Disease 2019 (COVID-19) pandemic, which may have influenced adolescents’ routines, eating habits, and psychosocial well-being.

Future research should prioritize longitudinal studies to establish the temporal relationship between dietary inflammatory potential and psychosocial functioning across adolescence. Incorporating inflammatory biomarkers and relevant early-life, familial, and psychological factors may further help elucidate the observed associations. Ultimately, intervention studies are needed to determine whether modifying dietary inflammatory potential translates into clinically meaningful improvements in psychosocial functioning.

## 5. Conclusions

The present findings suggest that a more pro-inflammatory dietary profile, as assessed using the DII-informed FFQ-derived inflammatory score, is associated with less favorable psychosocial functioning among adolescents, including higher total difficulties, greater conduct problems and hyperactivity/inattention, and lower prosocial behavior. However, these associations did not reach statistical significance in multiple-imputation analyses, warranting cautious interpretation of their robustness. These results add to growing evidence reporting associations between dietary inflammatory potential and psychosocial functioning during adolescence, particularly in externalizing domains. Further longitudinal and intervention studies are required to clarify the direction and causality of these relationships.

## Figures and Tables

**Table 1:** Descriptive characteristics of the study sample. Numbers of missing observations for each variable within the analytical sample are reported in the table footnotes/corresponding cells.

Variable	*N* = 674
Age (years)	14.0 (13.0, 15.0)
Sex	
Boys	289 (42.9%)
Girls	385 (57.1%)
FAS-III (score)	8.0 (7.0, 9.0)
BMI (kg/m^2^)	21.6 (19.3, 25.1)
Energy intake (kcal/day)	2582.0 (1954.5, 3460.5)
Sleep duration (hours/day)	8.3 (7.6, 8.8)
YAP-S Physical activity (score)	2.6 (2.2, 3.0)
YAP-S Sedentary behaviors (score)	3.4 (2.8, 4.0)
DII-informed FFQ-derived inflammatory score	0.3 (−5.4, 6.6)
SDQ	
Total difficulties (score)	11.0 (7.0, 16.0)
Emotional symptoms (score)	3.0 (1.0, 5.0)
Conduct problems (score)	2.0 (1.0, 3.0)
Hyperactivity/inattention (score)	4.0 (3.0, 6.0)
Peer relationship problems (score)	2.0 (1.0, 3.0)
Prosocial behavior (score)	8.0 (7.0, 9.0)
Externalizing problems (score)	6.0 (4.0, 8.0)
Internalizing problems (score)	5.0 (2.0, 8.0)

Available *n* varies by variable. Values are median (interquartile range) for continuous variables or *n* (percentage) for categorical variables. BMI, body mass index; DII, dietary inflammatory index; FAS-III, Family Affluence Scale-III; SDQ, Strengths and Difficulties Questionnaire; YAP-S, Spanish version of the Youth Activity Profile. Missing data within the analytical sample (*n* = 674): Age 0; Sex 0; FAS-III score 7 (1.0%); BMI 37 (5.5%); Energy intake 0; Sleep duration 8 (1.2%); YAP-S physical activity 9 (1.3%); YAP-S sedentary behavior 9 (1.3%); inflammatory score 0; all SDQ scores 0 (complete by analytical-sample definition). FAS-III item descriptives are reported in Supplementary Table S9.

**Table 2:** Association between the DII-informed FFQ-derived inflammatory score and different outcomes from the Strengths and Difficulties Questionnaire among Spanish adolescents.

SDQ Outcome	Unadjusted Model				Adjusted Model			
	B	95% CI	*p*	β	B	95% CI	*p*	β
Total difficulties	0.072	0.025 to 0.119	**0.003**	0.122	0.058	0.005 to 0.111	**0.031**	0.099
Emotional symptoms	0.008	−0.013 to 0.029	0.448	0.031	0.006	−0.018 to 0.029	0.637	0.022
Conduct problems	0.030	0.019 to 0.042	**<0.001**	0.179	0.025	0.011 to 0.039	**<0.001**	0.148
Hyperactivity/inattention	0.028	0.011 to 0.045	**0.001**	0.135	0.032	0.013 to 0.052	**0.001**	0.154
Peer relationship problems	0.008	−0.006 to 0.021	0.268	0.044	−0.0001	−0.016 to 0.016	0.990	−0.001
Prosocial behavior	−0.032	−0.045 to −0.019	**<0.001**	−0.162	−0.029	−0.045 to −0.014	**<0.001**	−0.149
Externalizing problems	0.059	0.034 to 0.084	**<0.001**	0.190	0.056	0.028 to 0.084	**<0.001**	0.181
Internalizing problems	0.016	−0.014 to 0.046	0.299	0.042	0.004	−0.030 to 0.038	0.803	0.011

The unadjusted models include participants with available data for the exposure and outcome variables (*n* = 674), whereas the adjusted models include participants with complete data for the exposure, outcome, and all covariates included in the respective model (*n* = 629). β, standardized beta coefficient; B, unstandardized beta coefficient; CI, confidence interval; *p*, *p*-value; SDQ, Strengths and Difficulties Questionnaire. Adjusted model includes: energy intake, age, sex, socioeconomic status, sleep duration, physical activity, sedentary behaviors, and body mass index. Robust regression (‘robustbase’ package; ‘lmrob’ function; ‘KS2014’ setting) was used. Significant results are shown in bold.

## Data Availability

The datasets are available from the corresponding author on reasonable request.

## Supplementary Materials

The following supporting information can be downloaded at: https://www.mdpi.com/article/10.3390/nu18183073/s1, Table S1. STROBE Statement for cross-sectional studies; Table S2. Missing data by variable (*N* = 1378); Table S3. Food items and DII-informed FFQ-derived inflammatory score weights; Table S4. Adjusted associations per 1-SD and IQR increase in inflammatory score; Table S5. Sensitivity analysis: quasi-Poisson regression (adjusted models); Table S6. Minimal vs full adjustment models (robust linear regression); Table S7. Multiple imputation vs complete-case comparison (MICE sensitivity); Table S8. MICE diagnostics: fraction of missing information (FMI) for the exposure coefficient; Table S9. FAS-III item descriptives in the analytical sample (*n* = 674); Table S10. Benjamini–Hochberg FDR correction across SDQ outcomes; Table S11. Sensitivity analyses: robust linear and OLS models; Table S12. Robust regression downweighting summary; Table S13. E-values for significant adjusted associations (corrected; B = unstandardized coefficient); Table S14. Comparison of included and excluded participants (missing-data sensitivity) (*N* = 1378); Table S15. Sensitivity analysis retaining inflammatory-score outliers (adjusted robust linear regression; Figure S1. Participant flow diagram; Figure S2. MICE convergence traces for chain means (diet-eligible sample after inflammatory-score outlier exclusion; m = 20, maxit = 10); Figure S3. Residual diagnostics—Total difficulties; Figure S4. Residual diagnostics—Emotional symptoms; Figure S5. Residual diagnostics—Conduct problems; Figure S6. Residual diagnostics—Hyperactivity/inattention; Figure S7. Residual diagnostics—Peer relationship problems; Figure S8. Residual diagnostics—Prosocial behavior; Figure S9: Residual diagnostics—Externalizing problems; Figure S10. Residual diagnostics—Internalizing problems.

## Abbreviations

BMI	Body mass index
CI	Confidence interval
COVID-19	Coronavirus Disease 2019
DII	Dietary inflammatory index
EHDLA	Eating Healthy and Daily Life Activities
FAS-III	Family Affluence Scale-III
FFQ	Food frequency questionnaire
IQR	Interquartile range
NHANES	National Health and Nutrition Examination Survey
SDQ	Strengths and Difficulties Questionnaire
YAP-S	Spanish Youth Activity Profile
