# Supplementary material for: Pro-Inflammatory Dietary Profiles Are Associated with Psychosocial Functioning in Spanish Adolescents: Findings from the EHDLA Study
Source: Nutrients. 2026 Sep 20;18(18):3073. doi: 10.3390/nu18183073 (PMC13610942; doi:10.3390/nu18183073)
Supplement: Supplementary file 1 [file nutrients-18-03073-s001.zip › nutrients-4516562-supplementary.pdf]

Supplementary Material
Table S1. STROBE Statement for cross-sectional studies

	Item No	Recommendation	Page No
Title and abstract	1	(a) Indicate the study's design with a commonly used term in the title or the abstract	1
		(b) Provide in the abstract an informative and balanced summary of what was done and what was found	1
Introduction			
Background/rationale	2	Explain the scientific background and rationale for the investigation being reported	2
Objectives	3	State specific objectives, including any prespecified hypotheses	2-3
Methods			
Study design	4	Present key elements of study design early in the paper	4
Setting	5	Describe the setting, locations, and relevant dates, including periods of recruitment, exposure, follow-up, and data collection	4
Participants	6	(a) Give the eligibility criteria, and the sources and methods of selection of participants	4
Variables	7	Clearly define all outcomes, exposures, predictors, potential confounders, and effect modifiers. Give diagnostic criteria, if applicable	5-6
Data sources/ measurement	8*	For each variable of interest, give sources of data and details of methods of assessment (measurement). Describe comparability of assessment methods if there is more than one group	5-6
Bias	9	Describe any efforts to address potential sources of bias	7-8
Study size	10	Explain how the study size was arrived at	5
Quantitative variables	11	Explain how quantitative variables were handled in the analyses. If applicable, describe which groupings were chosen and why	5-6
Statistical methods	12	(a) Describe all statistical methods, including those used to control for confounding	6-7
		(b) Describe any methods used to examine subgroups and interactions	6-7
		(c) Explain how missing data were addressed	7
		(d) If applicable, describe analytical methods taking account of sampling strategy	NA
		(e) Describe any sensitivity analyses	7
Results			
Participants	13*	(a) Report numbers of individuals at each stage of study—eg numbers potentially eligible, examined for eligibility, confirmed eligible, included in the study, completing follow-up, and analysed	5
		(b) Give reasons for non-participation at each stage	5
		(c) Consider use of a flow diagram	5
Descriptive data	14*	(a) Give characteristics of study participants (eg demographic, clinical, social) and information on exposures and potential confounders	8
		(b) Indicate number of participants with missing data for each variable of interest	8
Outcome data	15*	Report numbers of outcome events or summary measures	9-11
Main results	16	(a) Give unadjusted estimates and, if applicable, confounder-adjusted estimates and their precision (eg, 95% confidence	9-11

		interval). Make clear which confounders were adjusted for and why they were included	
		(b) Report category boundaries when continuous variables were categorized	9-11
		(c) If relevant, consider translating estimates of relative risk into absolute risk for a meaningful time period	10-11
Other analyses	17	Report other analyses done—eg analyses of subgroups and interactions, and sensitivity analyses	10-11
Discussion			
Key results	18	Summarise key results with reference to study objectives	13
Limitations	19	Discuss limitations of the study, taking into account sources of potential bias or imprecision. Discuss both direction and magnitude of any potential bias	14-15
Interpretation	20	Give a cautious overall interpretation of results considering objectives, limitations, multiplicity of analyses, results from similar studies, and other relevant evidence	13-14
Generalisability	21	Discuss the generalisability (external validity) of the study results	15
Other information			
Funding	22	Give the source of funding and the role of the funders for the present study and, if applicable, for the original study on which the present article is based	16

Table S2. Missing data by variable (N = 1378).

Variable	N (missing)	N (non-missing)	% (missing)
SDQ total difficulties (score)	640	738	46.4
Energy intake (kcal/day)	442	936	32.1
YAP-S physical activity (score)	296	1082	21.5
YAP-S sedentary behaviors (score)	296	1082	21.5
Sleep duration (min/day)	277	1101	20.1
FAS-III (score)	267	1111	19.4
Body mass index (kg/m^2)	117	1261	8.5

FAS-III, Family Affluence Scale-III; SDQ, Strengths and Difficulties Questionnaire; YAP-S, Youth Activity Profile.

Table S3. Food items and DII-informed FFQ-derived inflammatory score weights.

Food item	Energy (kcal/serving)	Weight	Direction	Weight rationale / source
1. Milk	134	0.1	Pro-inflammatory	Dairy (milk): neutral/mild pro-inflammatory in DII nutrient profile
2. Yogurt	79	-0.1	Anti-inflammatory	Fermented dairy (yogurt): anti-inflammatory (Shivappa et al., 2014 [20])
3. Chocolate	108	0.2	Pro-inflammatory	Added sugars/chocolate: pro-inflammatory (refined carbohydrate)
4. Breakfast cereals	133	0.3	Pro-inflammatory	Refined cereals: pro-inflammatory (high glycemic load)
5. Maria cookies	113	0.3	Pro-inflammatory	Refined bakery cookies: pro-inflammatory (refined grains, sugars)
6. Chocolate cookies	135	0.4	Pro-inflammatory	Refined bakery cookies with added fat/sugar: pro-inflammatory
7. Muffins, sponge cake	115	0.4	Pro-inflammatory	Pastries/muffins: pro-inflammatory (refined grains, saturated fat)
8. Ensaimada, donut, croissant	185	0.5	Pro-inflammatory	Ultra-processed pastries: pro-inflammatory (refined grains, trans/saturated fat)
9. Salad	17	-0.6	Anti-inflammatory	Vegetables (salad): anti-inflammatory (fiber, polyphenols)
10. Green beans, chard, spinach	46	-0.8	Anti-inflammatory	Green vegetables: anti-inflammatory (Shivappa et al., 2014 [20])
11. Side vegetables	28	-0.7	Anti-inflammatory	Vegetables: anti-inflammatory (Shivappa et al., 2014 [20])
12. Potatoes	138	0.1	Pro-inflammatory	Potatoes: mildly pro-inflammatory (starch load)
13. Legumes	205	-0.5	Anti-inflammatory	Legumes: anti-inflammatory (fiber, plant protein)
14. White rice	252	0.2	Pro-inflammatory	Refined grains (white rice): pro-inflammatory
15. Pasta	259	0.1	Pro-inflammatory	Refined grains (pasta): mildly pro-inflammatory
16. Soups and creams	12	-0.3	Anti-inflammatory	Soups: mildly anti-inflammatory (vegetable-based)
17. Eggs	79	0.1	Pro-inflammatory	Eggs: mildly pro-inflammatory
18. Chicken or turkey	247	0.0	Neutral	Poultry: neutral/mild pro-inflammatory
19. Beef, pork, lamb	336	0.4	Pro-inflammatory	Red meat: pro-inflammatory (Shivappa et al., 2014 [20])
20. Ground meat, sausage, hamburger	250	0.5	Pro-inflammatory	Processed meat: pro-inflammatory (Shivappa et al., 2014 [20])
21. White fish	123	-0.2	Anti-inflammatory	White fish: anti-inflammatory (omega-3 lean fish proxy)
22. Fatty fish	281	-0.4	Anti-inflammatory	Fatty fish: anti-inflammatory (omega-3; Shivappa et al., 2014 [20])
23. Shellfish	45	-0.1	Anti-inflammatory	Shellfish: mildly anti-inflammatory
24. Croquettes, empanadas, pizza	213	0.4	Pro-inflammatory	Ultra-processed mixed dishes: pro-inflammatory
25. Bread	121	0.2	Pro-inflammatory	Bread: mildly pro-inflammatory (refined grains)
26. Cured ham, sweet ham, processed meats	64	0.5	Pro-inflammatory	Processed meats: pro-inflammatory (Shivappa et al., 2014 [20])
27. Fresh cheese	47	0.0	Neutral	Cheese: neutral
28. Other cheeses	101	0.3	Pro-inflammatory	Cheese: mildly pro-inflammatory (saturated fat)
29. Citrus fruits	47	-0.5	Anti-inflammatory	Citrus fruits: anti-inflammatory (Shivappa et al., 2014 [20])
30. Other fruits	52	-0.5	Anti-inflammatory	Fruits: anti-inflammatory (Shivappa et al., 2014 [20])
31. Canned fruits	75	0.1	Pro-inflammatory	Canned fruits (added sugars): mildly pro-inflammatory
32. Natural fruit juices	90	-0.3	Anti-inflammatory	Natural fruit juice: anti-inflammatory (fruit-based)
33. Commercial fruit juices	102	0.2	Pro-inflammatory	Commercial fruit juice: mildly pro-inflammatory (free sugars)
34. Nuts	121	-0.4	Anti-inflammatory	Nuts: anti-inflammatory (Shivappa et al., 2014 [20])
35. Dairy desserts	145	0.2	Pro-inflammatory	Dairy desserts: mildly pro-inflammatory (added sugars)
36. Cream or chocolate cakes	385	0.5	Pro-inflammatory	Ultra-processed cakes: pro-inflammatory (refined grains, sugars, fat)
37. Candies	68	0.5	Pro-inflammatory	Confectionery: pro-inflammatory (added sugars)
38. Snack chips	161	0.5	Pro-inflammatory	Ultra-processed salty snacks: pro-inflammatory
39. Ice cream	207	0.3	Pro-inflammatory	Ice cream: mildly pro-inflammatory (added sugars, saturated fat)
40. Sugar-sweetened beverages	105	0.6	Pro-inflammatory	Sugar-sweetened beverages: pro-inflammatory (Shivappa et al., 2014 [20])
41. Low-calorie beverages	1	0.0	Neutral	Low-calorie beverages: neutral
42. Wine, sangria	83	-0.1	Anti-inflammatory	Alcohol (wine): mildly anti-inflammatory in DII literature
43. Beer	86	0.1	Pro-inflammatory	Alcohol (beer): mildly pro-inflammatory
44. Non-alcoholic beer	37	0.0	Neutral	Non-alcoholic beer: neutral
45. Distilled beverages	115	0.2	Pro-inflammatory	Distilled spirits: mildly pro-inflammatory

DII, dietary inflammatory index; FFQ, food frequency questionnaire; kcal, kilocalories. Note: Exact numerical weights were adopted from the previous EHDLA food-based inflammatory score (López-Gil et al., Eur J Nutr 2026 [21]). This weighting system has not been formally validated against inflammatory biomarkers or the canonical nutrient-based DII.

Table S4. Adjusted associations per 1-SD and IQR increase in inflammatory score.

**Outcome**	**N**	B (per 1 SD)	Lower 95% CI (1 SD)	Upper 95% CI (1 SD)	B (per IQR)	Lower 95% CI (IQR)	Upper 95% CI (IQR)	**p**
SDQ Total difficulties	629	0.596	0.054	1.138	0.677	0.061	1.292	0.031
SDQ Emotional symptoms	629	0.058	-0.185	0.301	0.066	-0.210	0.342	0.637
SDQ Conduct problems	629	0.257	0.116	0.399	0.292	0.132	0.452	<0.001
SDQ Hyperactivity/inattention	629	0.331	0.131	0.532	0.376	0.149	0.603	0.001
SDQ Peer relationship problems	629	-0.001	-0.165	0.163	-0.001	-0.187	0.184	0.990
SDQ Prosocial behavior	629	-0.300	-0.456	-0.143	-0.340	-0.518	-0.162	<0.001
SDQ Externalizing problems	629	0.573	0.285	0.862	0.651	0.324	0.978	<0.001
SDQ Internalizing problems	629	0.044	-0.302	0.390	0.050	-0.343	0.443	0.803

B, unstandardized regression coefficient; CI, confidence interval; IQR, interquartile range; SD, standard deviation; SDQ, Strengths and Difficulties Questionnaire.

Table S5. Sensitivity analysis: quasi-Poisson regression (adjusted models).

Outcome	N	Log rate ratio	Lower 95% CI	Upper 95% CI	p
SDQ Total difficulties	629	0.005	0.000	0.009	0.033
SDQ Emotional symptoms	629	0.002	-0.005	0.008	0.617
SDQ Conduct problems	629	0.012	0.005	0.020	0.002
SDQ Hyperactivity/inattention	629	0.007	0.003	0.012	0.002
SDQ Peer relationship problems	629	-0.001	-0.009	0.006	0.707
SDQ Prosocial behavior	629	-0.003	-0.005	-0.001	0.005
SDQ Externalizing problems	629	0.009	0.004	0.013	<0.001
SDQ Internalizing problems	629	0.000	-0.005	0.006	0.882

CI, confidence interval; RR, rate ratio; SDQ, Strengths and Difficulties Questionnaire.

Table S6. Minimal vs full adjustment models (robust linear regression).

Outcome	Model	N	B	Lower 95% CI	Upper 95% CI	p	Standardized β
SDQ Total difficulties	Minimal	667	0.075	0.029	0.121	0.001	0.127
SDQ Total difficulties	Full	629	0.058	0.005	0.111	0.031	0.099
SDQ Emotional symptoms	Minimal	667	0.010	-0.010	0.031	0.321	0.039
SDQ Emotional symptoms	Full	629	0.006	-0.018	0.029	0.637	0.022
SDQ Conduct problems	Minimal	667	0.030	0.018	0.042	<0.001	0.179
SDQ Conduct problems	Full	629	0.025	0.011	0.039	<0.001	0.148
SDQ Hyperactivity/inattention	Minimal	667	0.032	0.015	0.049	<0.001	0.151
SDQ Hyperactivity/inattention	Full	629	0.032	0.013	0.052	0.001	0.154
SDQ Peer relationship problems	Minimal	667	0.006	-0.007	0.020	0.361	0.036
SDQ Peer relationship problems	Full	629	0.000	-0.016	0.016	0.990	-0.001
SDQ Prosocial behavior	Minimal	667	-0.030	-0.043	-0.017	<0.001	-0.151
SDQ Prosocial behavior	Full	629	-0.029	-0.045	-0.014	<0.001	-0.149
SDQ Externalizing problems	Minimal	667	0.061	0.037	0.086	<0.001	0.197
SDQ Externalizing problems	Full	629	0.056	0.028	0.084	<0.001	0.181
SDQ Internalizing problems	Minimal	667	0.016	-0.013	0.045	0.291	0.041
SDQ Internalizing problems	Full	629	0.004	-0.030	0.038	0.803	0.011

B, unstandardized regression coefficient; CI, confidence interval; SDQ, Strengths and Difficulties Questionnaire; β, standardized regression coefficient.

Table S7. Multiple imputation vs complete-case comparison (MICE sensitivity).

Outcome	Analysis	N	B	95% CI	p
Total difficulties	Complete-case (lmrob)	629	0.06	0.01 to 0.11	0.031
Emotional symptoms	Complete-case (lmrob)	629	0.01	-0.02 to 0.03	0.637
Conduct problems	Complete-case (lmrob)	629	0.03	0.01 to 0.04	<0.001
Hyperactivity/inattention	Complete-case (lmrob)	629	0.03	0.01 to 0.05	0.001
Peer relationship problems	Complete-case (lmrob)	629	-0.0001	-0.02 to 0.02	0.990
Prosocial behavior	Complete-case (lmrob)	629	-0.03	-0.04 to -0.01	<0.001
Externalizing problems	Complete-case (lmrob)	629	0.06	0.03 to 0.08	<0.001
Internalizing problems	Complete-case (lmrob)	629	0.004	-0.03 to 0.04	0.803
Total difficulties	MI pooled OLS (m=20)	928	0.04	-0.01 to 0.09	0.099
Emotional symptoms	MI pooled OLS (m=20)	928	0.003	-0.04 to 0.04	0.895
Conduct problems	MI pooled OLS (m=20)	928	0.02	-0.01 to 0.05	0.116
Hyperactivity/inattention	MI pooled OLS (m=20)	928	0.02	-0.02 to 0.05	0.351
Peer relationship problems	MI pooled OLS (m=20)	928	-0.001	-0.02 to 0.02	0.945
Prosocial behavior	MI pooled OLS (m=20)	928	-0.03	-0.05 to 0.0003	0.053
Externalizing problems	MI pooled OLS (m=20)	928	0.04	-0.001 to 0.08	0.054
Internalizing problems	MI pooled OLS (m=20)	928	0.005	-0.04 to 0.05	0.845

B, unstandardized regression coefficient; CI, confidence interval; MICE, multivariate imputation by chained equations; SDQ, Strengths and Difficulties Questionnaire.

Table S8. MICE diagnostics: fraction of missing information (FMI) for the exposure coefficient.

Outcome	B	95% CI	p	FMI	lambda
Total difficulties	0.050	-0.024 to 0.125	0.181	0.578	0.562
Emotional symptoms	-0.000	-0.039 to 0.039	0.990	0.703	0.686
Conduct problems	0.021	-0.005 to 0.048	0.111	0.678	0.661
Hyperactivity/inattention	0.020	-0.012 to 0.052	0.210	0.655	0.638
Peer relationship problems	0.006	-0.017 to 0.029	0.631	0.511	0.496
Prosocial behavior	-0.025	-0.050 to -0.001	0.045	0.555	0.539
Externalizing problems	0.041	0.001 to 0.081	0.043	0.589	0.573
Internalizing problems	0.012	-0.036 to 0.060	0.612	0.580	0.564

B, unstandardized regression coefficient; CI, confidence interval; FMI, fraction of missing information; MICE, multivariate imputation by chained equations; lambda, proportion of total variance attributable to missingness. Pooled OLS estimates from m = 20 imputations (maxit = 10) among diet-eligible participants after outlier exclusion (N = 928). Auxiliary variables: Type_of_family, Height, Weight, and FAS_III items 1–6. MICE (m = 20; maxit = 10; N = 928) included analysis variables (DII-informed FFQ-derived inflammatory score, SDQ outcomes, and covariates) plus auxiliary variables: Type_of_family, Height, Weight, FAS_III_1, FAS_III_2, FAS_III_3, FAS_III_4, FAS_III_5, FAS_III_6. Predictive mean matching was used for continuous variables. Convergence was inspected via chain-mean traces (Figure S2). Fraction of missing information (FMI) for the exposure coefficient ranged from 0.511 to 0.703 across SDQ outcomes (median 0.585), which is consistent with the substantial incomplete-case burden in this sensitivity sample and supports cautious interpretation of the MI estimates.

Table S9. FAS-III item descriptives in the analytical sample (n = 674).

Item	N	N missing	Median	IQR	Mean	SD
Family car/van (0-2)	667	7	2	1	1.64	0.54
Own bedroom (0-1)	667	7	1	0	0.84	0.36
Computers/tablets (0-3)	667	7	3	1	2.33	0.82
Bathrooms (0-3)	667	7	2	1	2.06	0.69
Dishwasher (0-1)	667	7	1	0	0.76	0.43
Family holidays abroad last 12 months (0-3)	667	7	0	1	0.5	0.88

FAS-III, Family Affluence Scale-III; IQR, interquartile range; SD, standard deviation.

Table S10. Benjamini–Hochberg FDR correction across SDQ outcomes.

Outcome	Adjusted p	Adjusted β	FDR-adjusted p
SDQ Total difficulties	0.031	0.10	0.050
SDQ Emotional symptoms	0.637	0.02	0.850
SDQ Conduct problems	<0.001	0.15	<0.001
SDQ Hyperactivity/inattention	0.001	0.15	0.002
SDQ Peer relationship problems	0.990	0.00	0.990
SDQ Prosocial behavior	<0.001	-0.15	<0.001
SDQ Externalizing problems	<0.001	0.18	<0.001
SDQ Internalizing problems	0.803	0.01	0.917

FDR, false discovery rate; SDQ, Strengths and Difficulties Questionnaire; β, adjusted regression coefficient.

Table S11. Sensitivity analyses: robust linear and OLS models.

Outcome	N	Robust linear model				Ordinary least square model			
		B	Lower 95% CI	Upper 95% CI	p	B	Lower 95% CI	Upper 95% CI	p
SDQ Total difficulties	629	0.06	0.01	0.11	0.031	0.06	0.01	0.11	0.028
SDQ Emotional symptoms	629	0.01	-0.02	0.03	0.637	0.01	-0.02	0.03	0.608
SDQ Conduct problems	629	0.03	0.01	0.04	<0.001	0.02	0.01	0.04	0.001
SDQ Hyperactivity/inattention	629	0.03	0.01	0.05	0.001	0.03	0.01	0.05	0.002
SDQ Peer relationship problems	629	0.00	-0.02	0.02	0.990	0.00	-0.02	0.01	0.728
SDQ Prosocial behavior	629	-0.03	-0.04	-0.01	<0.001	-0.03	-0.04	-0.01	0.004
SDQ Externalizing problems	629	0.06	0.03	0.08	<0.001	0.05	0.03	0.08	<0.001
SDQ Internalizing problems	629	0.00	-0.03	0.04	0.803	0.00	-0.03	0.04	0.852

B, unstandardized regression coefficient; CI, confidence interval; OLS, ordinary least squares; SDQ, Strengths and Difficulties Questionnaire.

Table S12. Robust regression downweighting summary.

Outcome	N	N (weight < 0.5)	% (weight < 0.5)	N (weight < 0.2)	% (weight < 0.2)	Min (weight)	Median (weight)
SDQ Total difficulties	629	8	1.3	0	0.0	0.291	1
SDQ Emotional symptoms	629	2	0.3	0	0.0	0.411	1
SDQ Conduct problems	629	17	2.7	3	0.5	0.171	1
SDQ Hyperactivity/inattention	629	2	0.3	0	0.0	0.362	1
SDQ Peer relationship problems	629	6	1.0	0	0.0	0.259	1
SDQ Prosocial behavior	629	20	3.2	9	1.4	0.073	1
SDQ Externalizing problems	629	3	0.5	0	0.0	0.277	1
SDQ Internalizing problems	629	7	1.1	0	0.0	0.320	1

SDQ, Strengths and Difficulties Questionnaire.

Table S13. E-values for significant adjusted associations (corrected; B = unstandardized coefficient).

Outcome	B	Lower 95% CI	Upper 95% CI	E-value (point)	E-value (CI)
SDQ Total difficulties	0.06	0.01	0.11	1.01	1.00
SDQ Conduct problems	0.03	0.01	0.04	1.01	1.01
SDQ Hyperactivity/inattention	0.03	0.01	0.05	1.01	1.01
SDQ Prosocial behavior	-0.03	-0.04	-0.01	1.01	1.01
SDQ Externalizing problems	0.06	0.03	0.08	1.02	1.01

B, unstandardized regression coefficient; CI, confidence interval; SDQ, Strengths and Difficulties Questionnaire.

Table S14. Comparison of included and excluded participants (missing-data sensitivity) (N = 1378).

Group	N	Age, median	Age, IQR	Girls, %	BMI, median	BMI, IQR	FAS-III, median	FAS-III, IQR	Energy intake, median	Energy intake, IQR
Included (adjusted complete-case)	629	14	2	56.9	21.6	5.8	8.0	2	2592.7	1498.0
Excluded: incomplete covariates	45	14	2	60.0	19.5	6.0	8.0	2	2106.6	1337.8
Excluded: incomplete SDQ	254	14	3	50.0	21.5	6.3	8.5	3	2588.4	1492.8
Excluded: incomplete dietary/energy data	442	15	4	41.0	21.9	6.4	8.0	3	-	-

BMI, body mass index; FAS-III, Family Affluence Scale-III; IQR, interquartile range; SDQ, Strengths and Difficulties Questionnaire

Table S15. Sensitivity analysis retaining inflammatory-score outliers (adjusted robust linear regression).

Outcome	Analysis	N	B	95% CI	p
Total difficulties	Primary (outliers excluded; lmrob)	629	0.058	0.005 to 0.111	0.031
Total difficulties	Retaining outliers (lmrob)	634	0.061	0.019 to 0.103	0.004
Emotional symptoms	Primary (outliers excluded; lmrob)	629	0.006	-0.018 to 0.029	0.637
Emotional symptoms	Retaining outliers (lmrob)	634	0.015	-0.004 to 0.034	0.115
Conduct problems	Primary (outliers excluded; lmrob)	629	0.025	0.011 to 0.039	<0.001
Conduct problems	Retaining outliers (lmrob)	634	0.017	0.006 to 0.028	0.003
Hyperactivity/inattention	Primary (outliers excluded; lmrob)	629	0.032	0.013 to 0.052	0.001
Hyperactivity/inattention	Retaining outliers (lmrob)	634	0.031	0.015 to 0.046	<0.001
Peer relationship problems	Primary (outliers excluded; lmrob)	629	-0.000	-0.016 to 0.016	0.990
Peer relationship problems	Retaining outliers (lmrob)	634	0.002	-0.011 to 0.015	0.772
Prosocial behavior	Primary (outliers excluded; lmrob)	629	-0.029	-0.045 to -0.014	<0.001
Prosocial behavior	Retaining outliers (lmrob)	634	-0.013	-0.025 to 0.000	0.051
Externalizing problems	Primary (outliers excluded; lmrob)	629	0.056	0.028 to 0.084	<0.001
Externalizing problems	Retaining outliers (lmrob)	634	0.046	0.024 to 0.069	<0.001
Internalizing problems	Primary (outliers excluded; lmrob)	629	0.004	-0.030 to 0.038	0.803
Internalizing problems	Retaining outliers (lmrob)	634	0.016	-0.010 to 0.043	0.229

B, unstandardized regression coefficient; CI, confidence interval; lmrob, robust linear regression. Primary models exclude inflammatory-score outliers (3×IQR rule; n = 8). Sensitivity models retain all diet-complete participants with complete covariates.

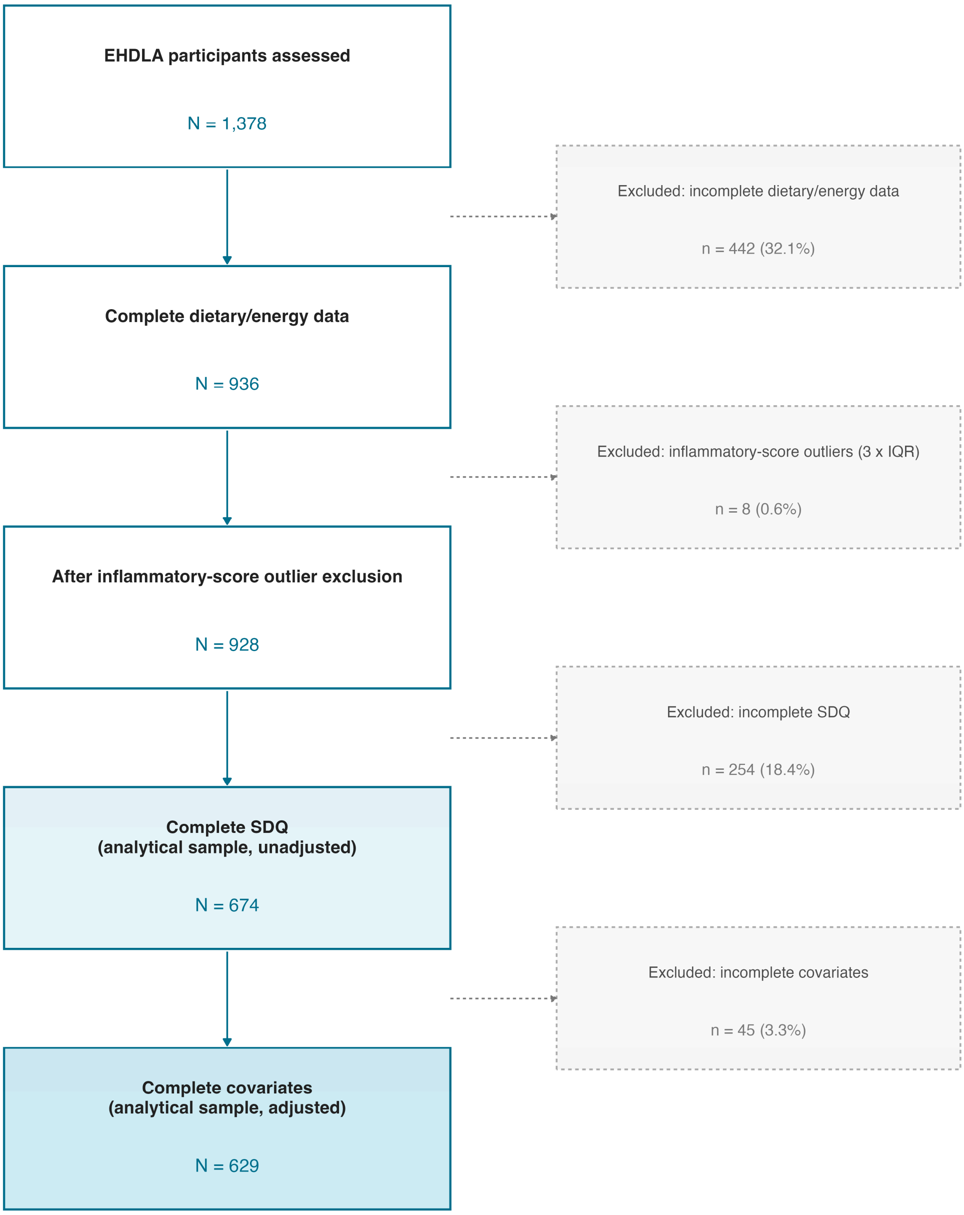

Figure S1. Participant flow diagram. DII, dietary inflammatory index; EHDLA, Eating Healthy and Daily Life Activities; SDQ, Strengths and Difficulties Questionnaire.

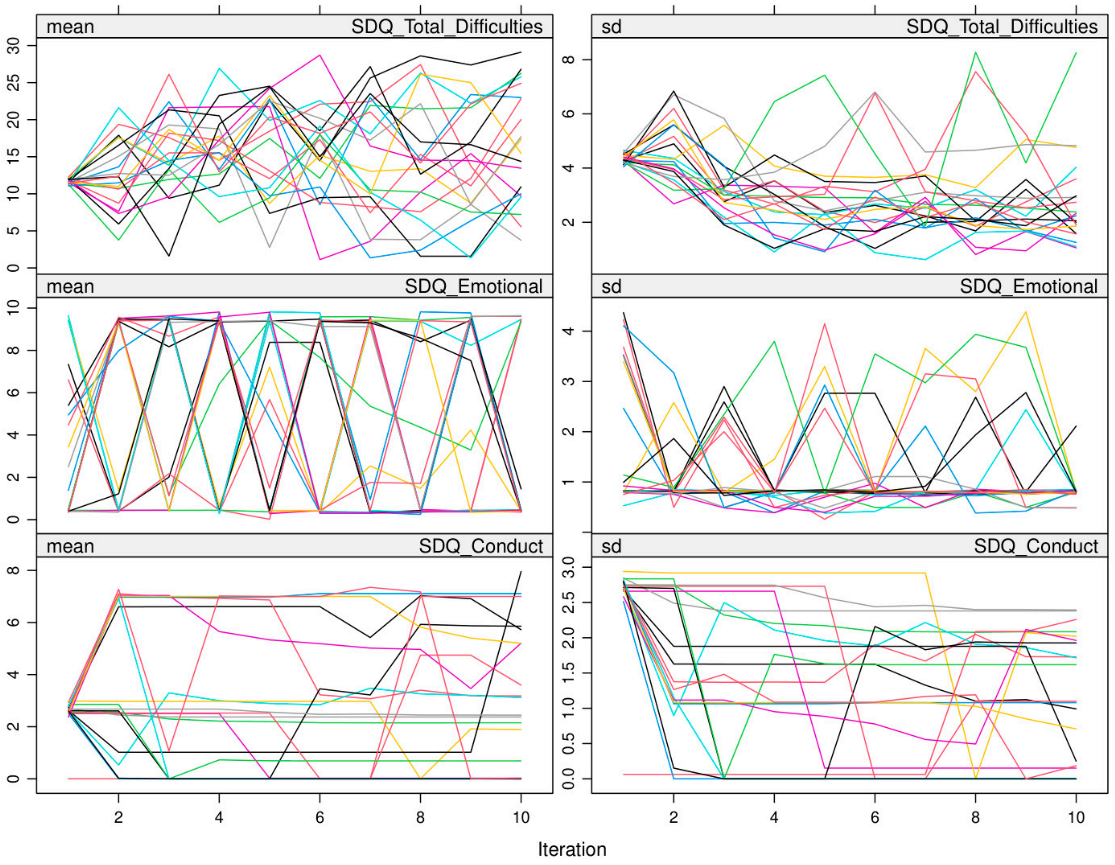

Figure S2. MICE convergence traces for chain means (diet-eligible sample after inflammatory-score outlier exclusion; m = 20, maxit = 10). MICE, multivariate imputation by chained equations.

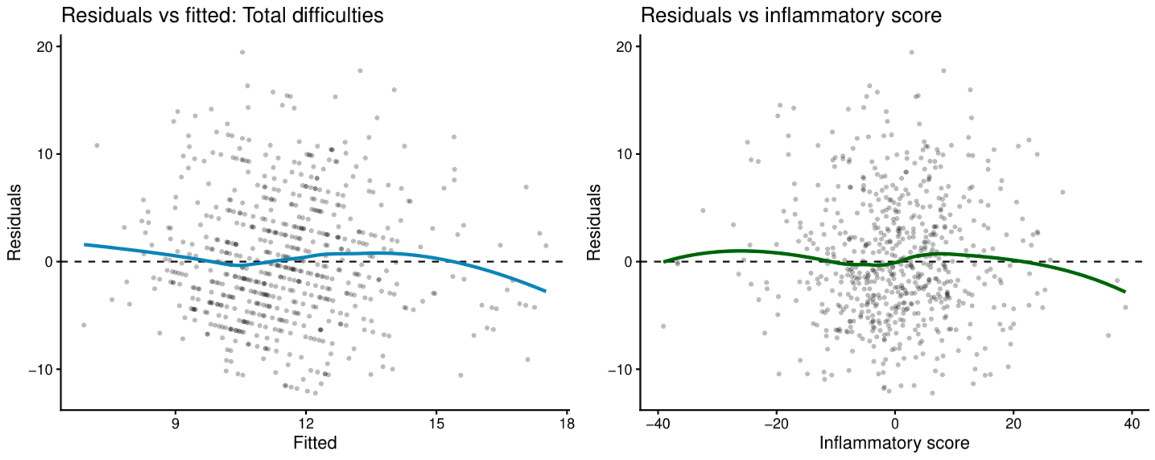

Figure S3. Residual diagnostics—Total difficulties.

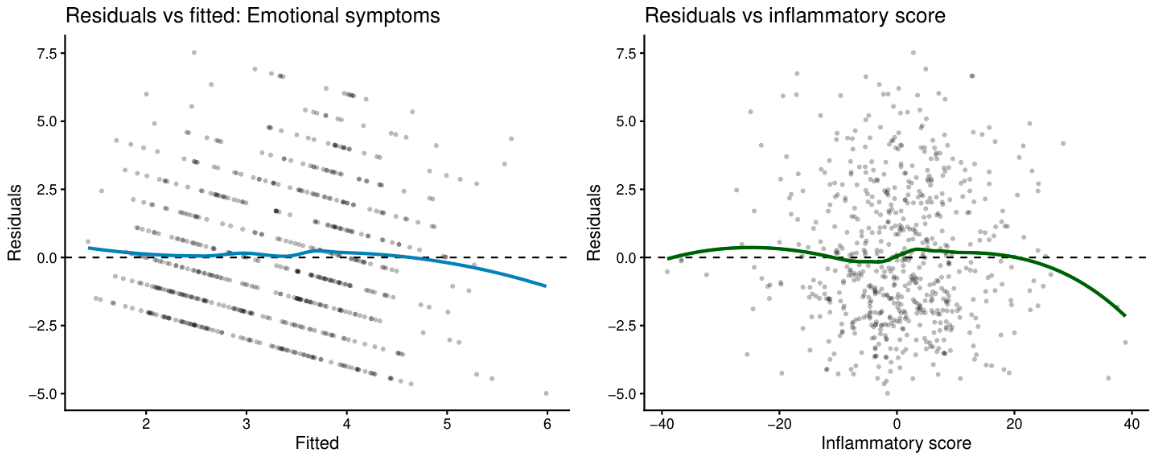

Figure S4. Residual diagnostics—Emotional symptoms.

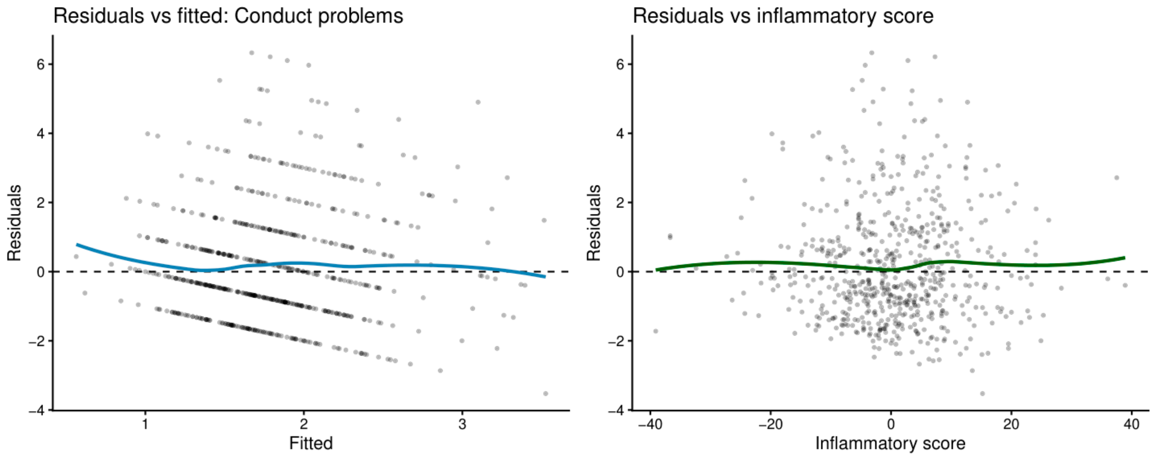

Figure S5. Residual diagnostics—Conduct problems.

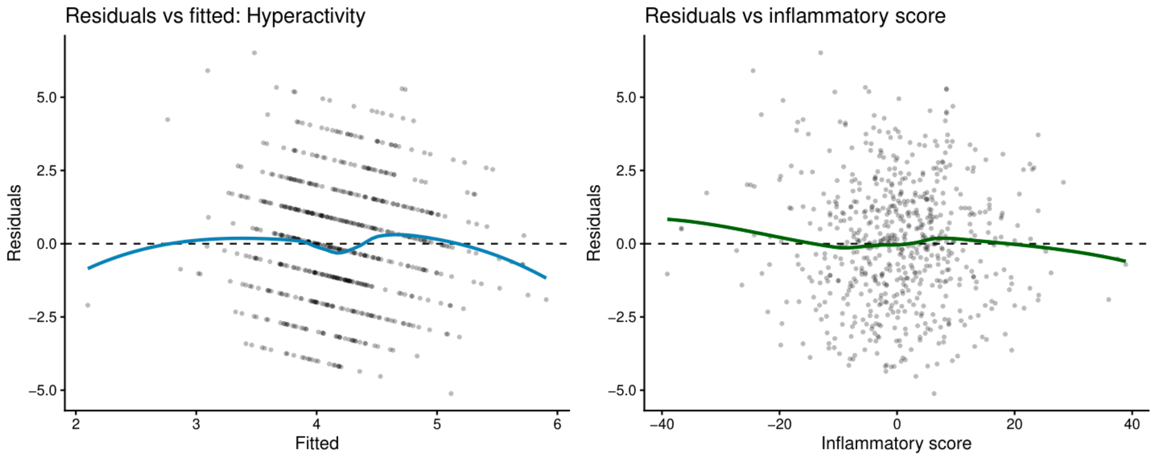

Figure S6. Residual diagnostics—Hyperactivity/inattention.

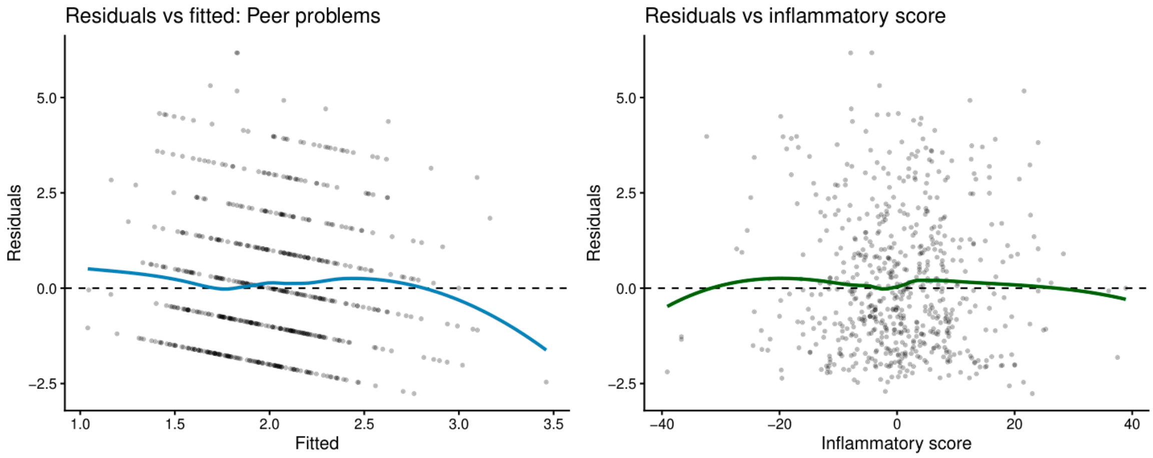

Figure S7. Residual diagnostics—Peer relationship problems.

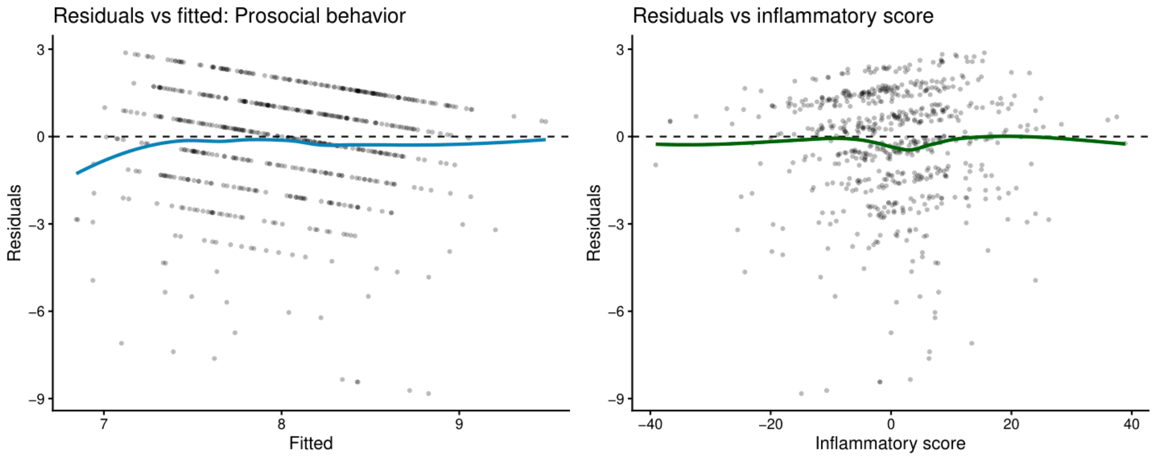

Figure S8. Residual diagnostics—Prosocial behavior.

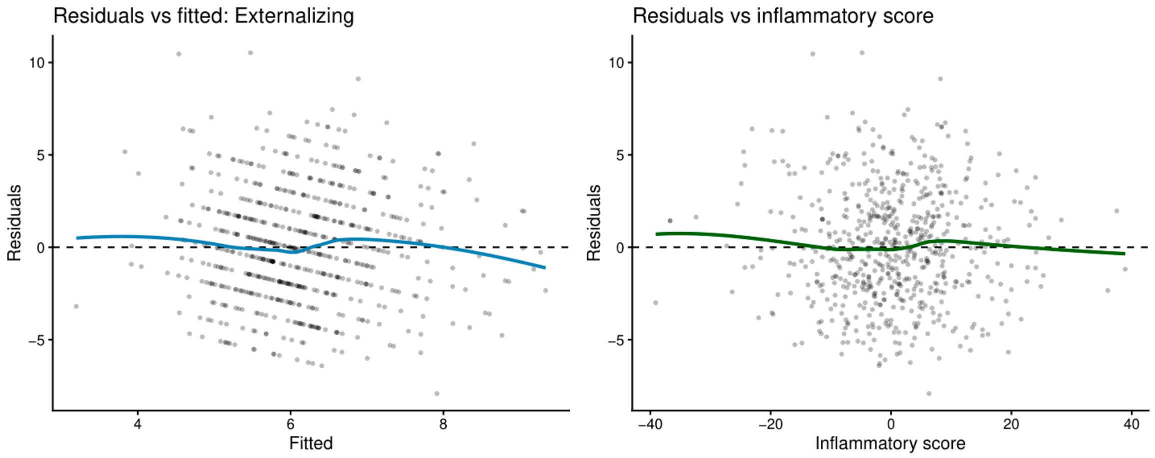

Figure S9. Residual diagnostics—Externalizing problems.

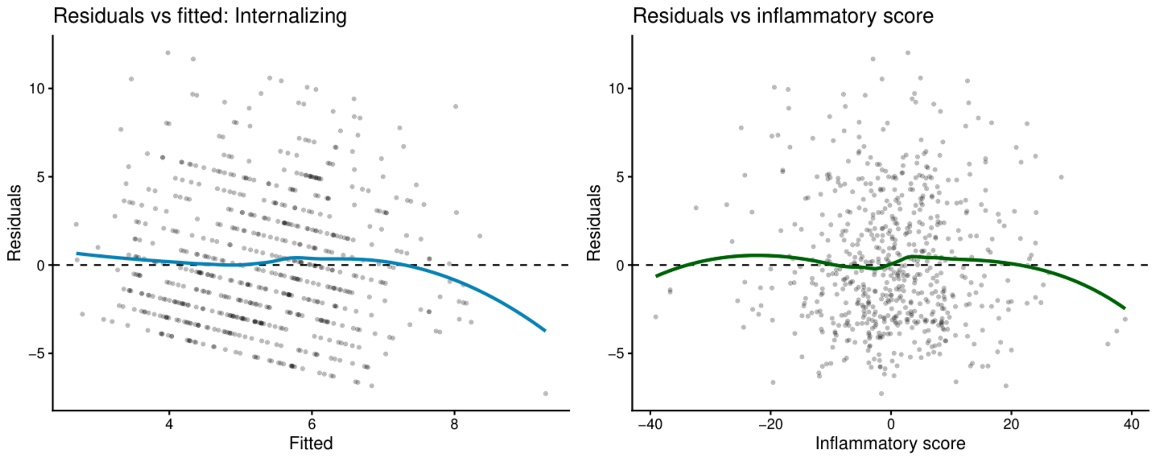

Figure S10. Residual diagnostics—Internalizing problems
